# Immunological Basis for Rapid Progression of Diabetes in Older NOD Mouse Recipients Post BM-HSC Transplantation

**DOI:** 10.1371/journal.pone.0128494

**Published:** 2015-05-28

**Authors:** Nan Wang, Narendiran Rajasekaran, Tieying Hou, Claudia Macaubas, Elizabeth D. Mellins

**Affiliations:** Program in Immunology, Division of Human Gene Therapy, Department of Pediatrics, Stanford University School of Medicine, Stanford, California, United States of America; University of Cincinnati College of Medicine, UNITED STATES

## Abstract

Type I diabetes (T1D), mediated by autoreactive T cell destruction of insulin-producing islet beta cells, has been treated with bone marrow-derived hematopoietic stem cell (BM-HSC) transplantation. Older non-obese diabetic (NOD) mice recipients (3m, at disease-onset stage) receiving syngeneic BM-HSC progressed more rapidly to end-stage diabetes post-transplantation than younger recipients (4-6w, at disease-initiation stage). FACS analyses showed a higher percentage and absolute number of regulatory T cells (Treg) and lower proportion of proliferating T conventional cells (Tcon) in pancreatic lymph nodes from the resistant mice among the younger recipients compared to the rapid progressors among the older recipients. Treg distribution in spleen, mesenteric lymph nodes (MLN), blood and thymus between the two groups was similar. However, the percentage of thymic Tcon and the proliferation of Tcon in MLN and blood were lower in the young resistants. These results suggest recipient age and associated disease stage as a variable to consider in BM-HSC transplantation for treating T1D.

## Introduction

Type I diabetes (T1D) is a disorder of insulin production, resulting from the destruction of pancreatic beta-cells by auto-reactive T cells. The rapidly rising incidence of T1D worldwide, thought to be the result of the interplay of genetic, epigenetic, and environmental factors, represents an increasing global public health burden. T1D induces long-term complications, such as kidney disease, blindness, heart attacks, strokes and amputations that still lack efficient therapies. The mainstay insulin replacement therapy does not parallel normal insulin kinetics and fails to restore normoglycemia in most T1D patients, who experience an increased risk of hypoglycemia, failure to control postprandial hyperglycemia and unpredictable diurnal glucose fluctuations [1]. Pancreas transplantation is accompanied with surgical and immunosuppressive complications, whereas islet transplantation requires high-level technique [2].

Among the therapies for T1D, bone marrow transplantation (BMT) has the potential to interrupt and reverse the autoimmune process prior to the development of long-term complications [3]. BMT also induces host tolerance to pancreas or islet cell transplants from the same donor. Previous studies showed that BMT from mismatched major histocompatibility complex (MHC) or non-MHC donors blocks T1D in NOD mouse recipients [4]. T1D in leukemia patients reversed after receiving allogeneic BMT from healthy donors [5]. Even syngeneic or autogeneic BMT helps to block may attenuate T1D progression. For example, in one study, syngeneic BMT slightly delayed T1D onset in NOD mice, although disease incidence was similar with or without the transplantation [6]. Autologous nonmyeloablative transplantation of hematopoietic stem cell (HSC) together with cyclophosphamide and rabbit antithymocyte globulin treatment improves islet function and induces insulin independence in most newly diagnosed T1D patients [7]. The results from this human trial corroborate previous studies showing that T1D is linked more closely with HSC than with their derivatives [3].

Hematopoietic stem cell transplantation (HSCT) has been traditionally used as a therapy for hematologic malignancies in young patients due to the higher morbidity and mortality in the elderly [8]. Autologous HSCT trials recruited only newly diagnosed T1D patients [7]. In animal experiments, the preferred age of NOD mice receiving BM or HSC transplantation from allogeneic or syngeneic donors is usually below 8w [6,9–14] when T1D is still at the initiation or peri-insulitis stage [15]. Here, we analyzed the immunological features of two groups of recipients at different ages and associated disease stages receiving same donor BM-HSC, but with significantly different progression of T1D. The results may provide some explanations for the above-mentioned preference for young/early disease stage recipients.

## Results

### Rapid progression of T1D in older NOD recipients post syngeneic BM-HSC transplantation

**Fig 1:**
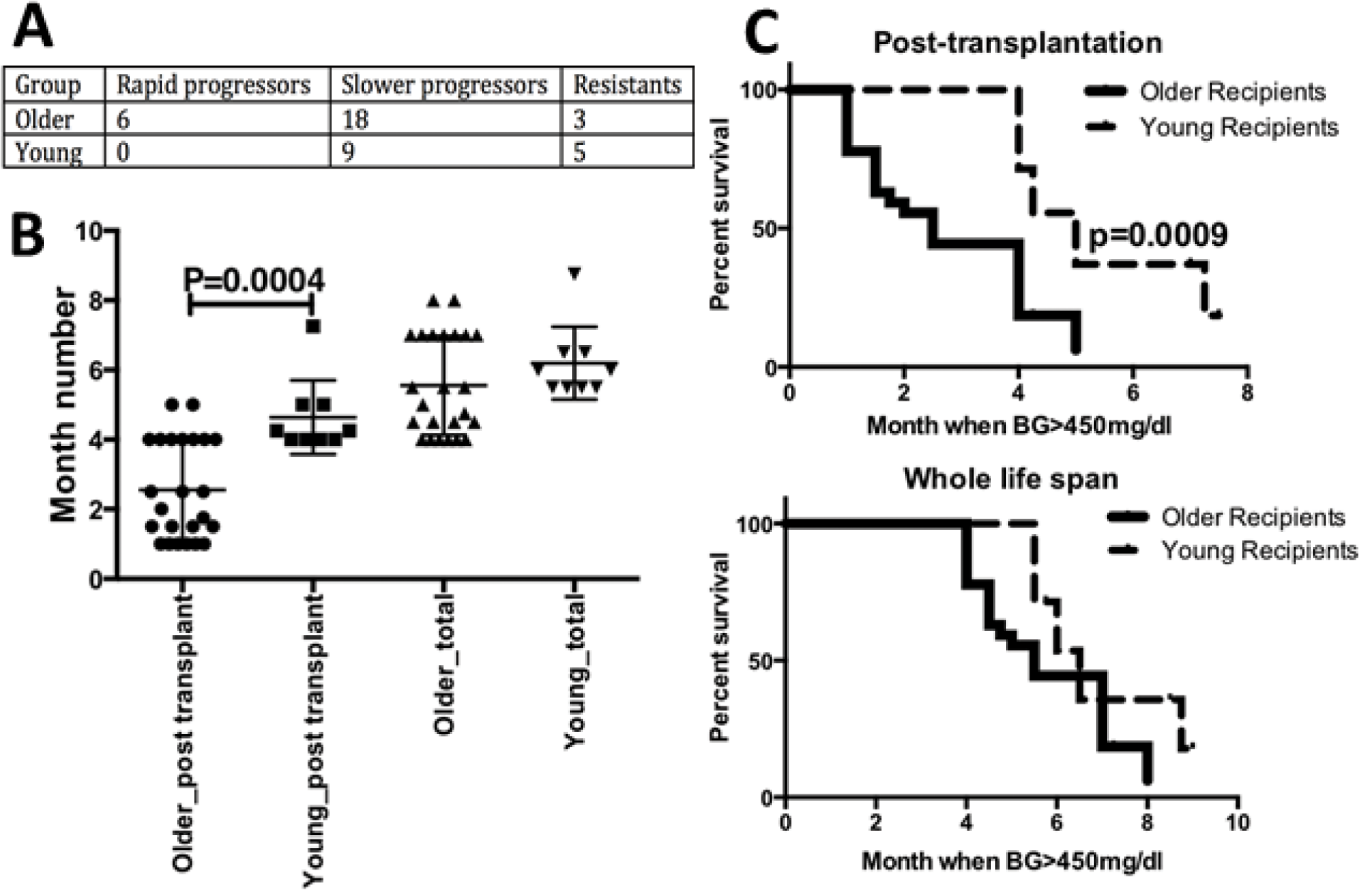
Rapid T1D progression in older NOD recipients post syngeneic BM-HSC transplantation. A: Statistical summary of disease progression post syngeneic BM-HSC transplantation. Two groups of mice, i.e. young recipients (age 4-6w, at initiation stage) and older recipients (age 3m, at advance stage) respectively, were irradiated and received mixed BM-HSCs harvested from 3-5m NOD donors (50% with high blood glucose). T1D progression was categorized by the month number post-transplantation when recipients developed blood glucose >450mg/dl, i.e. ≤1m as rapid progressors, 1-7m as slower progressors, and those with normal glucose as resistants. Chi-square test was applied giving a P value as 0.0503. B. Month number when mice had end-stage T1D or blood glucose >450mg/dl. Rapid and slower progressors from panel A in each age group were combined and their T1D progression was calculated by the month number when blood glucose >450mg/dl counting either post-transplantation time (1^st^ and 2^nd^ columns) or the whole life span (3^rd^ and 4^th^ columns). Unpaired 2-tailed T test was used to compare the young vs. older recipients under same category (post-transplantation or life span). Only the difference of T1D progression between young vs. older recipients post-transplantation was significant with P <0.05. C. Survival curves for young vs. older recipients post-transplantation (top) or based on life span (bottom). All mice, regardless of progression subgroup, were included. The log-rank (Mantel-Cox) test was used to analyze survival rates. Those mice had blood glucose >450mg/dl were counted as deceased. P value is shown only when the difference was statistically significant, i.e. P <0.05. When whole life span was analyzed, older recipients had a shorter life span than the young recipients (P = 0.0786, Fig 1C bottom). Although this P value is modestly higher than the cut-off for statistical significance, P = 0.05, there is a trend toward significant survival benefit in younger recipients.

To evaluate progression of T1D in syngeneic BM-HSC NOD recipients with different ages/disease stages at transplantation, two groups of mice, with age 4–6w (young recipients) and 3m (older recipients) respectively, were irradiated and received mixed BM-HSCs harvested from 3–5m NOD donors. The selection criteria for the age of recipients were based on the natural disease progression, i.e. 4–6w mice were at the disease initiation stage with peri-insulitis, whereas the 3m mice were at the disease onset stage with destructive insulitis [15]. About 50% of the donors had high glucose (i.e. blood glucose >250mg/dl), whereas all recipients had normal glucose at the time of transplantation. Results from 6 individual experiments were combined, as shown in Fig 1A. We categorized the T1D progression based on the time (month number) when recipients developed blood glucose >450mg/dl post-transplantation, i.e. ≤1m as rapid progressors, 1–7m as slower progressors and normal glucose as resistants. We observed more progressors among the older recipients than those among the young recipients (Fig 1A). Chi-square test was applied to test the relationship between the age of recipients and the speed of T1D progression. The P value was 0.0503, suggesting a correlation between the age of recipients at transplantation and the progression of diabetes thereafter.

We then used the unpaired 2-tailed *t* test to compare the progression speed among the young vs. older recipients. Here, we combined rapid and slower progressors from same age group together and excluded the resistants. The time points (month number) when the recipients got end stage T1D or had blood glucose >450mg/dl were counted. As shown in Fig 1B, when counted as time after transplantation, T1D progressed significantly faster in older recipients than in young group (P <0.01). However, when their actual ages from birth were calculated, the older recipients got the final stage disease only slightly earlier (by age) than the young recipients, and the difference did not reach statistical significance. To further confirm that diabetes progressed more rapidly in older group compared to young group animals post-transplantation, but did not alter life span, we did survival analysis for the two groups. All sub-groups, i.e. rapid and slower progressors as well as resistants, were included; mice with blood glucose >450mg/dl were counted as deceased. Using log-rank (Mantel-Cox) test, we found results similar to the T test results, i.e. the older group got end-stage diabetes significantly faster post-transplantation (p = 0.0009, Fig 1C top). Older recipients had a shorter life span than younger recipients, although this was not statistically significant (P = 0.0786, Fig 1C bottom).

### Higher abundance of Treg in pancreatic lymph nodes (PLN) from young recipients compared to older recipients

To investigate the mechanism(s) underlying the rapid progression of T1D in older recipients post-transplantation, we compared the immunological phenotypes of the rapid progressors in the older recipient group (6 mice with blood glucose >450mg/dl within 1m post-transplantation) and the resistants from the young recipient group (5 mice with normal blood glucose (<200mg/dl) at sacrifice, typically 7.5m post-transplantation). In the following experiments, these older progressors and young resistants are termed as older and young, respectively. The groups were designated by the age of the mice at transplantation, not at harvesting. We performed FACS analyses of immune cells from various immune organs.

**Fig 2:**
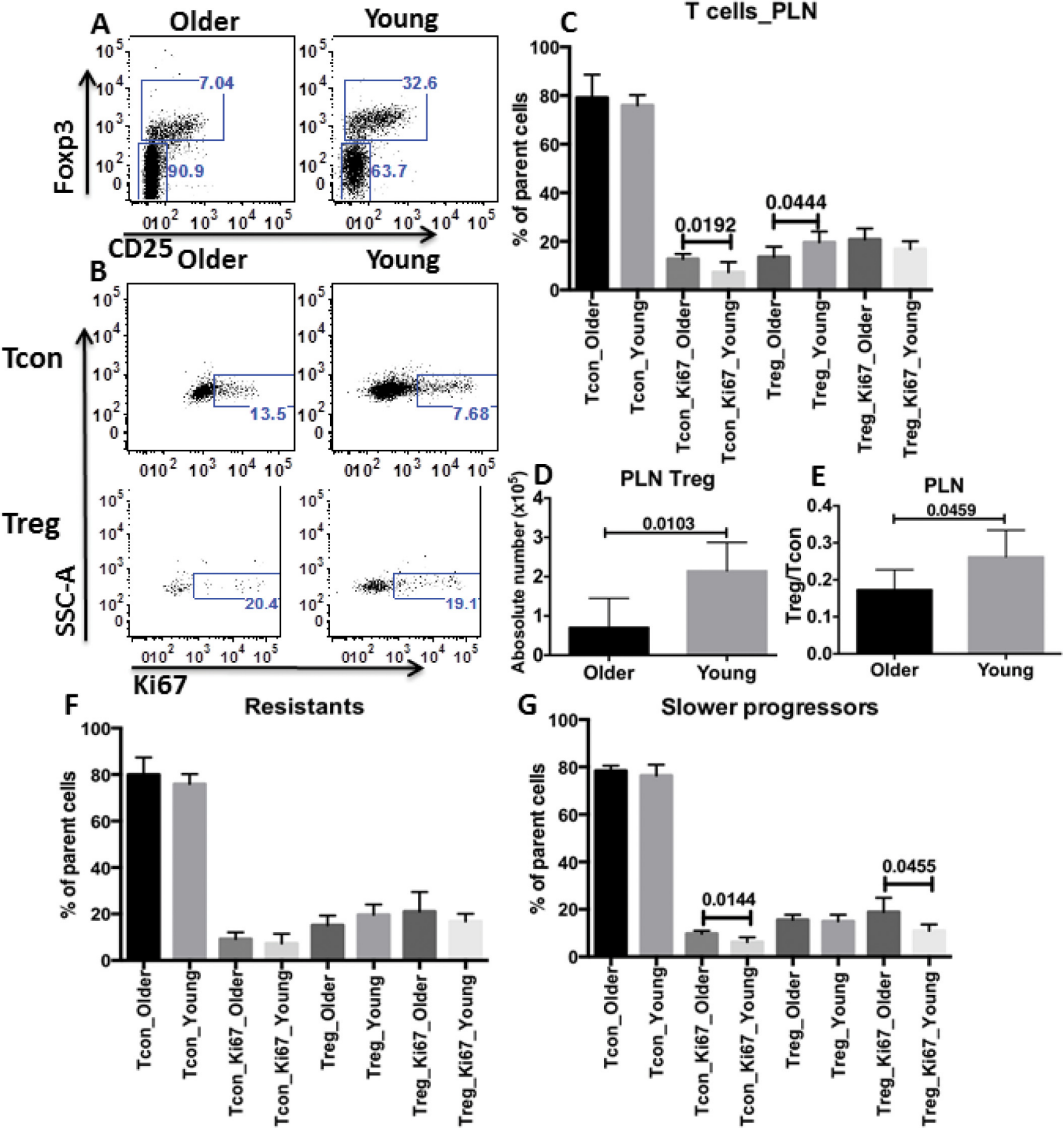
Higher abundance of Treg and lower proliferation of Tcon in PLN from young recipients. A. Representative FACS data of PLN Tcon and Treg distribution of one rapid progressor from older recipients (indicated as older) and that of one resistant from young recipients (as young). B. Representative FACS data showed the proliferation level of PLN Tcon (top panels) and Treg (bottom panels) of one older rapid progressor (indicated as older) and one young resistant (as young). C. Statistical analyses of the percentage and proliferation of PLN Tcon and Treg from older rapid progressors (as older, n = 6) vs. young resistants (as young, n = 5). D. Statistical analyses of the absolute number of PLN Treg in older rapid progressors (as older) vs. young resistants (as young). The absolute numbers were calculated as: total PLN number (by hemo-cytometer) times percentage of Treg among total PLN cells (by FACS). E. Comparison of the ratios of percentage of Treg to percentage of Tcon in PLN between the older rapid progressors (as older) and young resistants (as young). F. The percentage and proliferation of Tcon and Treg in 3 older resistants vs. those parameters in 5 young resistants. G. The percentage and proliferation of Tcon and Treg in slower progressors that reached end-stage diabetes at 4m post-transplantation (n = 4 and 5 for young and older recipients respectively). For panels C through G, unpaired 2-tailed T test was applied for comparing values between the older rapid progressors and the young resistants (panels C to E), between the young and the older resistants (panel F) or between the young and the older 4m progressors (panel G). Only those with statistical significance or P <0.05 were labeled. “Parent cells” in panels C, F and G indicates the immediate upstream gating of the corresponding subset.

Because conventional T cells (Tcon) and regulatory T cells (Treg) from PLN are crucial in mediating and controlling T1D respectively [16], we first measured the abundance and proliferation of these 2 subsets of CD4^+^ T cells. CD4^+^CD25^−^Foxp3^-^ and CD4^+^ Foxp3^+^ were used to define Tcon and Treg, respectively. The cell division marker, Ki67, was used to demonstrate the level of cell proliferation [17]. The percentage of PLN Treg was significantly higher in the young vs. older group (Fig 2A and 2C). The difference in absolute number of PLN Treg in the young vs. older group was even larger (Fig 2D). There was no significant difference between the percentages of PLN Tcon from older and young groups (Fig 2C). Accordingly, the ratio of Treg to Tcon (Treg/Tcon) was significantly higher in the young vs. older group (Fig 2E). Notably, the proliferation of Tcon was lower in the young vs. older group (Fig 2B and 2C). Due to the essential roles played by PLN Tcon and Treg in the pathogenesis of T1D, the lower abundance of Treg and higher proliferation of Tcon in PLN may at least partially explain the rapid progression of T1D in older recipients.

To exclude the possibility that the PLN Tcon and Treg differences we observed between the young resistants and older progressors relate only to the disease stage of T1D at harvesting, instead of to the age of recipients at BM-HSC transplantation, we compared Tcon and Treg status in young vs. older recipients that progressed similarly after transplantation (i.e. both groups got end stage of T1D or were resistant at same time point post-transplantation). Specifically, we compared the percentage and proliferation level of PLN Tcon and Treg between young and older resistants (n = 5 and n = 3 respectively, Fig 2F) as well as between slower progressors that got end stage disease at 4m post-transplantation in young and older recipients (n = 4 and n = 5 respectively, Fig 2G). Among the young recipients, there were no rapid progressors, i.e. none got end stage disease within 1m post-BMT, so these could not be compared to older rapid progressors. Trends toward higher percentage of Tcon and lower percentage of Treg as well as higher proliferation of both Treg and Tcon were observed in older resistant group compared to the young resistant group, but these differences were not statistically significant in the small number of animals compared (Fig 2F). For the slower (4m) progressors, the percentages of Tcon and Treg were comparable across the age groups (Fig 2G). However, the cells of older progressors proliferated more than those of young progressors, with statistically significant differences for both Tcon and Treg. The higher proliferation of Tcon in older progressors may reflect more effective function of Treg in young progressors or more resistant Tcon in older progressors. The higher proliferation of Treg may be a compensatory mechanism in older progressors. We conclude that the differences of Tcon and Treg status between young and older recipients do not only reflect the disease stage difference. In addition, we measured the percentage and absolute number of PLN Tcon and Treg from non-transplanted NOD mice at the corresponding age and similar blood glucose level (i.e. 4–6w and 3m with normal blood glucose (NG) as the controls for transplant recipients at transplant as well as 3–4m and 7–8m with normal glucose (NG) or high blood glucose (HG, glucose >450mg/dl) as controls for rapid progressors and resistants post-transplantation). There appears to be a small, but not statistically significant (P >0.05), changes in abundance of Treg (increase) and Tcon (decrease) as mice age. No statistical significance was found in either Treg or Tcon populations between age-matched NG and HG groups (S1 Fig).

### Lower proliferation of MLN and blood Tcon in young vs. older recipients, with similar percentages of Treg

**Fig 3:**
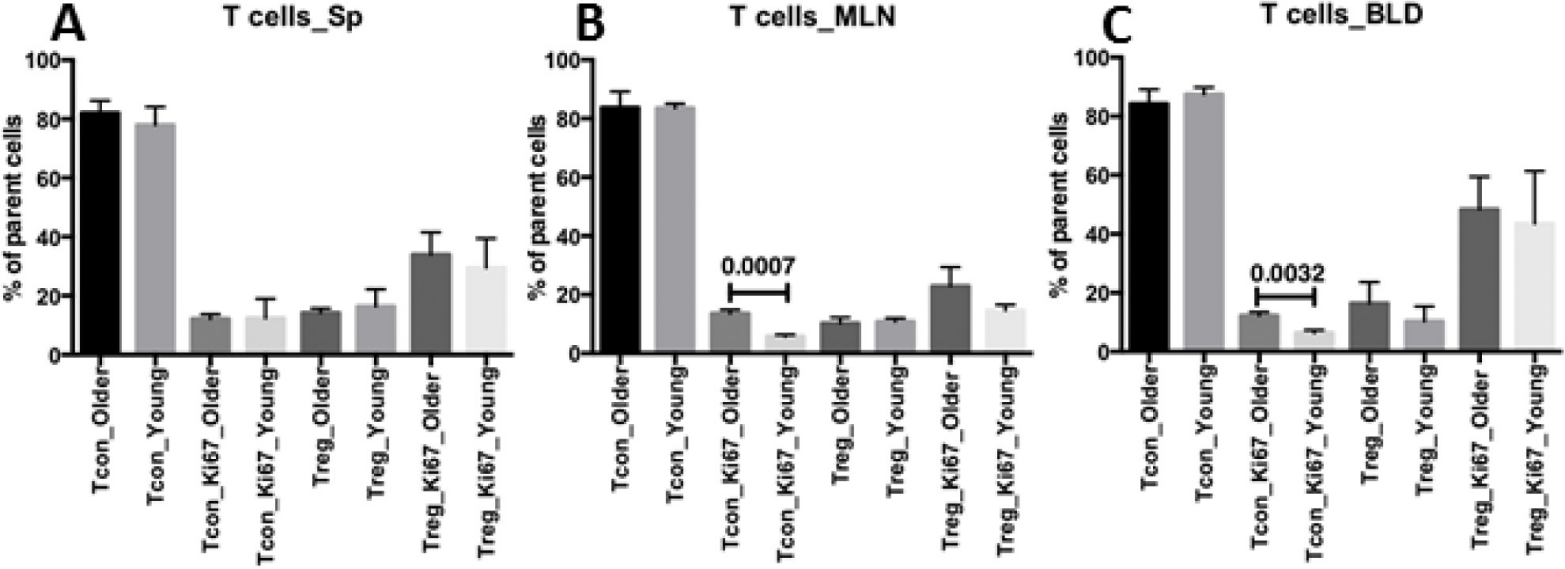
Lower proliferation of MLN and blood Tcon from young recipients. Samples of spleens (Sp, panel A), mesenteric lymph nodes (MLN, panel B) and blood (BLD, panel C) harvested from individual older rapid progressors (as older, n = 6) and young resistants (as young, n = 5) were analyzed with unpaired 2-tailed T test for the percentage and proliferation of Tcon and Treg. Only those with statistical significance or P <0.05 are labeled.

To see if the trend of higher abundance of Treg and lower proliferation of Tcon in PLN of young recipients extends to other peripheral immune organs or in the circulation, we explored the percentage and proliferation of Tcon and Treg from spleen, mesenteric lymph nodes (MLN) and blood. Consistent with PLN Tcon cells, Tcon from both MLN and blood proliferated less in young recipients than in older recipients (Fig 3B and 3C). However, no differences between Treg percentages from MLN and blood in young and older groups were observed (Fig 3B and 3C). Splenocytes from both young and older groups showed similar cell distribution and proliferation of Tcon and Treg (Fig 3A).

### Lower percentage of thymic Tcon from young vs. older recipients

**Fig 4:**
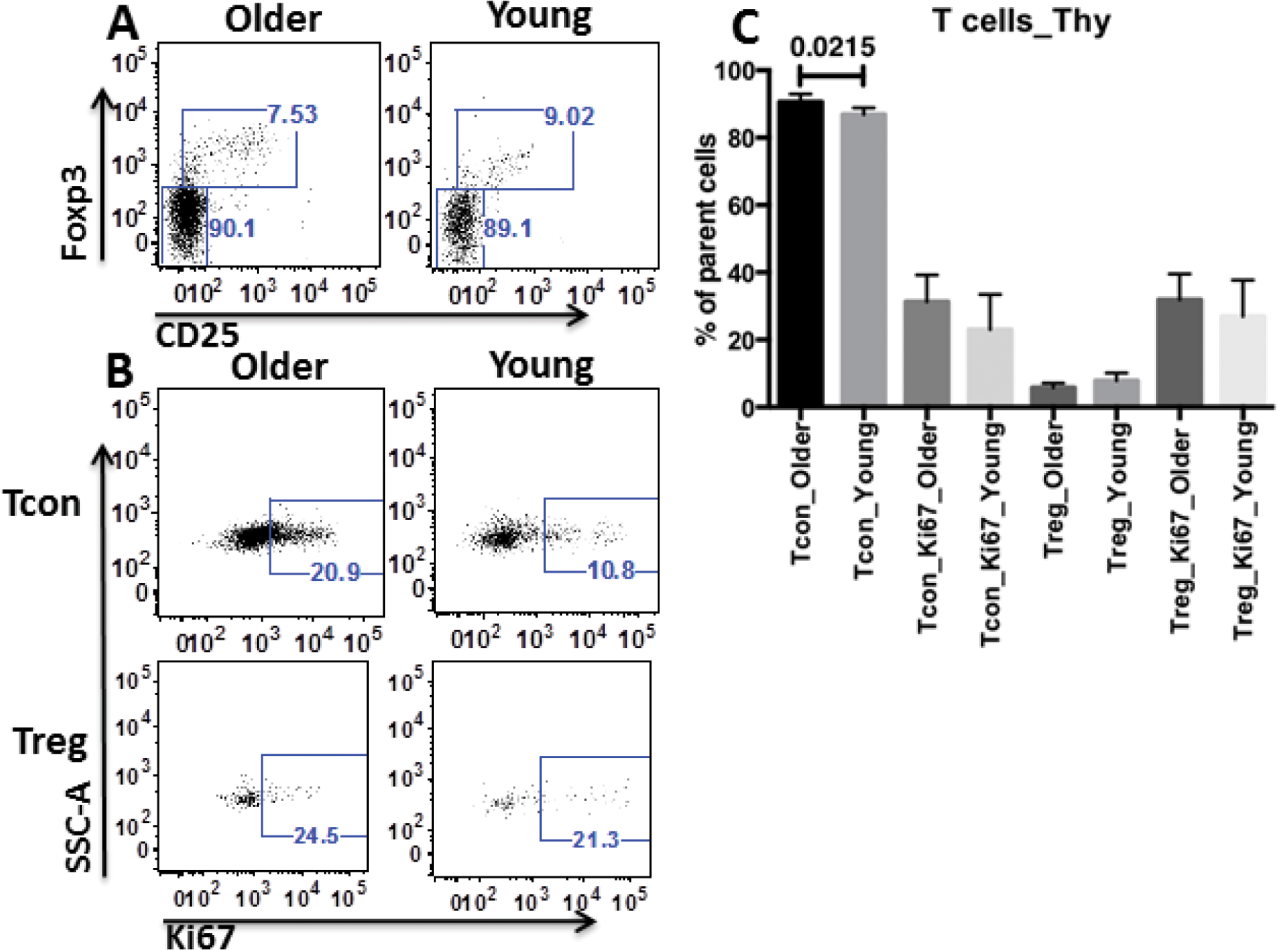
Lower percentage of thymic Tcon from young recipients. A. Representative FACS data of thymic Tcon and Treg distribution of one older rapid progressor (as older) and that of one young resistant (as young). B. Representative FACS data showed the proliferation level of thymic Tcon (top panels) and Treg (bottom panels) of one older rapid progressor (as older) and one young resistant (as young). C. Statistical analyses of the percentage and proliferation of thymic Tcon and Treg from older rapid progressors (as older, n = 6) vs. young resistants (as young, n = 5). For panel C, unpaired 2-tailed T test was applied. Only the difference in percentage of thymic Tcon between young resistants and older rapid progressors was statistically significant at P <0.05.

To investigate whether the difference in PLN Treg between young and older groups originated in the thymus, the cell distribution and proliferation levels were measured in thymocytes from those recipients. A modest, but statistically significant, reduction in the percentage of Tcon was observed in young recipients compared to older recipients (Fig 4A and 4C). The proliferation of both Tcon and Treg as well as the percentage of Treg were comparable in young and older recipients (Fig 4). Thus, the higher abundance of Treg did not appear to originate in the thymus.

### Similar pro-inflammatory cytokine production in spleen from young and older recipients

**Fig 5:**
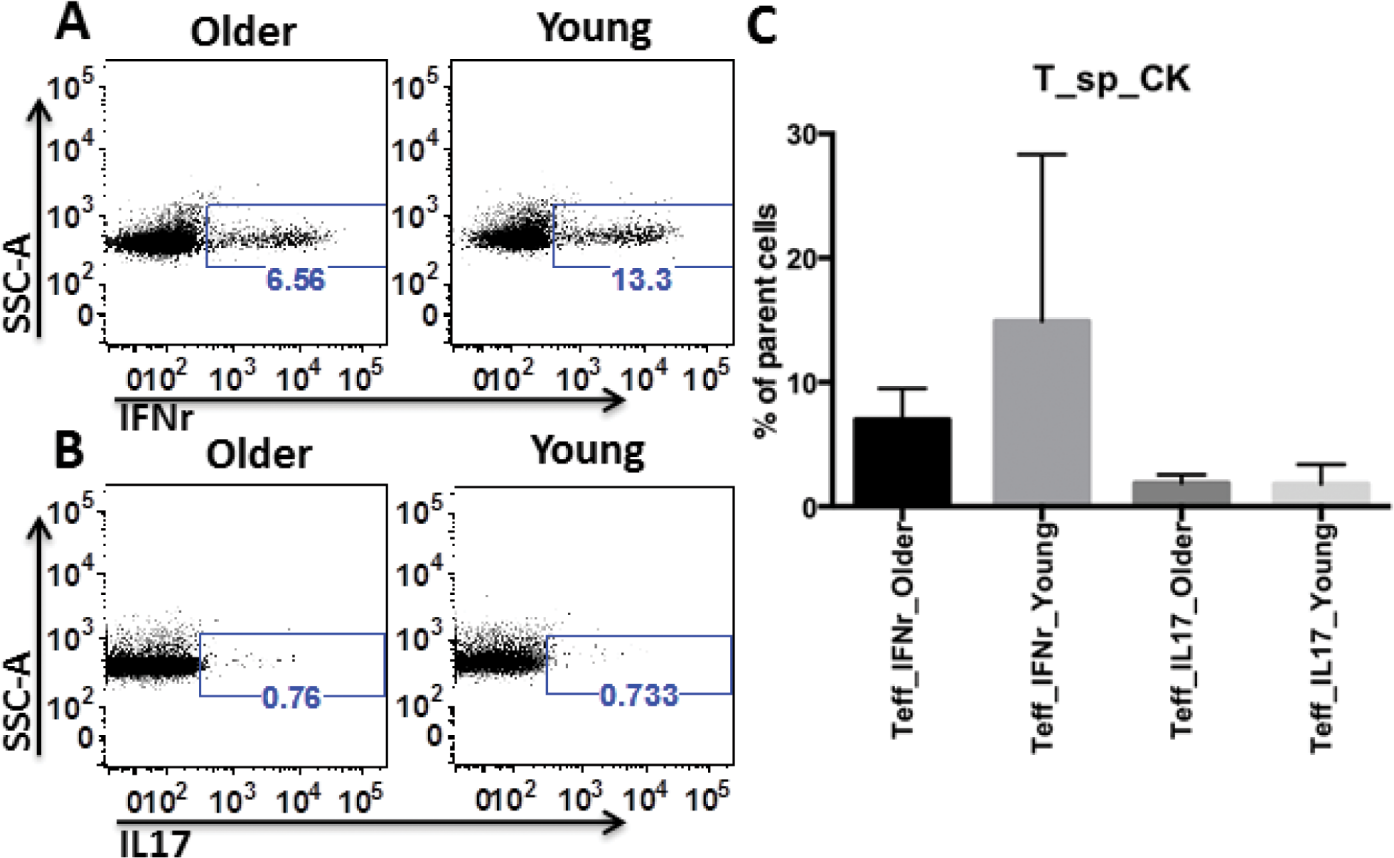
Comparable IFNγ and IL-17 production by splenocytes from young resistants and older progressors. Representative FACS data of IFNγ (A) and IL-17 (B) production by splenic Tcon from an older rapid progressor (as older) and a young resistant (as young). C. Statistical analyses of IFNγ and IL-17 production from older progressors (indicated as older, n = 6) and young resistants (as young, n = 5). Unpaired 2-tailed T test was used, and no significant difference between the two groups was found.

Th1 type cytokine IFN gamma (IFNγ) is a key player in destroying insulin-secreting beta cells, and the more recently discovered, pro-inflammatory cytokine IL-17 also plays an important role in T1D development, both in NOD mice and in T1D patients [18,19]. To measure IFNγ and IL-17 responses in young and older recipients, splenocytes were harvested, stimulated, fixed, permeabilized, and stained with appropriate antibodies. There were no statistically significant differences in the percentages of splenic Tcon producing IFNγ or IL-17 between young and older recipients (Fig 5). However, the mean proportion of IFNγ^+^ Tcon cells was higher in young recipients than in older recipients (Fig 5C), consistent with previous reports of reduced proportions of peripheral blood CD4^+^ cells producing IFNγ in T1D patients compared to controls and subjects at risk, perhaps due to their migration to pancreas [20]. No significant difference was found in either proportions or cytokine production in CD8^+^ T cells from thymus or peripheral immune organs between young and older recipients (S2 Fig).

### Comparable levels of I-A^g7^ in young and older recipients

**Fig 6:**
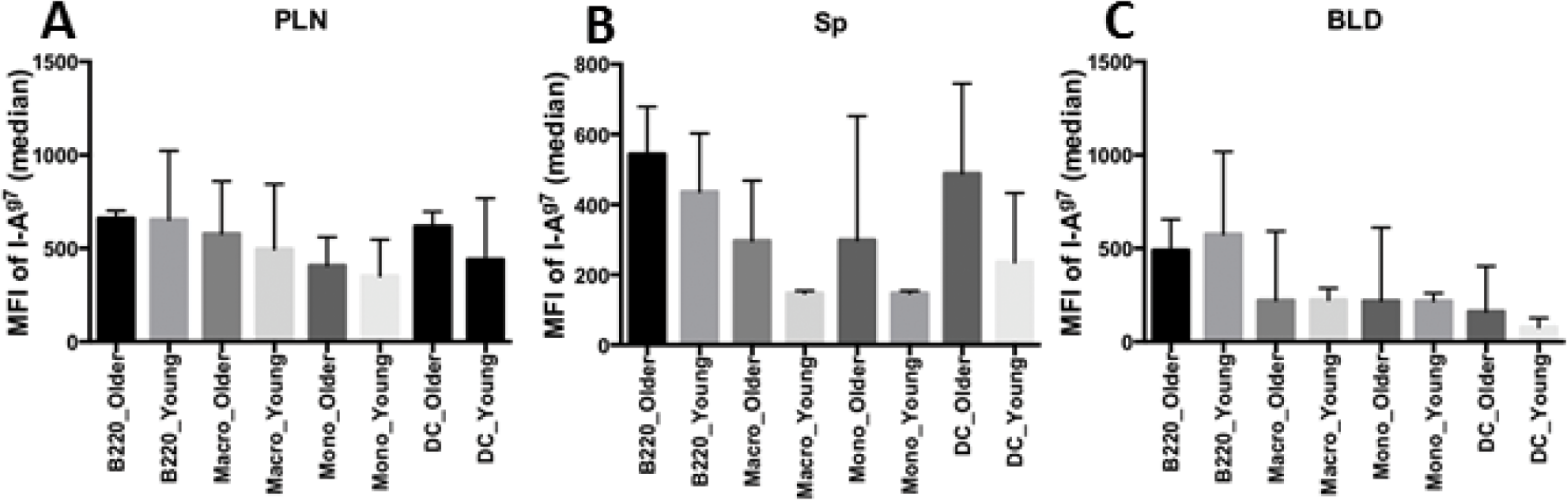
Similar I-A^g7^ level of various antigen presenting cell (APC) types from young resistants and older progressors. MFI of I-A^g7^ from PLN (A), spleen (B) and blood (C) was summarized and analyzed by unpaired 2-tailed T test. B220 was used as a marker for B cells. Macro, Mono, and DC stand for macrophages, monocytes and dendritic cells respectively. No significant difference between young resistants and older progressors was found.

I-A^g7^, the MHC class II allele expressed by NOD mice, is relatively unstable and has similar structural characteristics as its human counterpart, DQ8 [21]. I-A^g7^ has lower than normal affinity for CLIP (class II-associated invariant chain peptides), a fragment of its chaperone, invariant chain [22]. Enhancement of I-A^g7^-CLIP interaction increases I-A^g7^ abundance and reduces the presentation of diabetogenic mimotopes [23]. To determine if there are any differences in I-A^g7^ level between the older and young recipients, cells from PLN, spleen and blood were stained with the antibodies to markers of various antigen presenting cell lineages (B cells, macrophages, monocytes and DC) and I-A^g7^. FACS analysis showed no significant differences in I-A^g7^ levels between young and older recipients in the four APC cell types we tested in PLN, spleen or blood (Fig 6).

## Discussion

BM or HSC transplantation is a possible curative treatment for leukemia, solid tumors, and metabolic diseases. However, many problems prevent its use in elderly patients, including intrinsic pattern of disease features with poorer prognosis, more frequent comorbidities, higher incidence of acute and chronic graft-versus-host disease (GVHD), more intolerance to the conditioning regimen and longer recovery time to normal neutrophil and platelet level [8]. Animal experiments using mouse recipients at different ages also demonstrated enhanced GVHD [24] and reduced homing efficiency of donor HSC [25] in old recipients. Moreover, reduced engraftment at lower irradiation doses was observed in adult mouse recipients compared to neonatal recipients receiving the same irradiation dose. No donor-derived cells were found at the target organ (brain) in adult recipients even with successful blood engraftment [26]. In the current study, we focused on the effect of recipients’ age-related disease stage on the results of BM-HSC transplantation.

BM-HSC transplantation in animal models stimulated clinical trials of autologous HSC transplantation for the treatment of various severe, refractory, autoimmune diseases including T1D. As indicated by the standard transplantation regimen mentioned above, both animal experiments in NOD mice and clinical trials in T1D patients used recipients at very young age or initiation stage of disease [6,7,9–14]. Accordingly, using NOD mice at different ages or disease stages, i.e young mice at 4–6w with peri-insulitis and older mice at 3m with destructive insulitis, we observed more rapid T1D progression in older recipients post-transplantation, although both recipient groups had normal glucose when they received BM-HSC from same syngeneic donors (Fig 1). More advanced disease stage in older compared to young recipients at the time of transplantation may be one of the main reasons for this faster progression. Using gene-targeted mice at different ages without pre-existing islet injury as recipients may give an even clearer understanding of the differences in response to transplant.

Here, we explored the immunological characteristic(s) underlying this age/disease stage difference. Results revealed higher abundance of Treg in PLN, the center of T1D pathogenesis [27], from young recipients who maintained normal glucose than from older recipients who progressed rapidly to the end-stage disease (Fig 2). Treg, which dampen the activities effector T cells (Teff) or Tcon, are essential for preserving immune homeostasis and preventing autoimmune diseases, including T1D. The key role of Treg function in blocking T1D development was demonstrated by experimental reduction/depletion and by genetic deficiency of these cells and adoptive transfer [28,29]. Initially, reduced frequency and/or function of Treg were reported in both T1D patients [30] and NOD mice [31]. Later studies disputed those results by demonstrating normal Treg number and function in T1D patients [32] and NOD mice [33], due to newly identified markers and more consistent research conditions [34]. Instead, the defect in Treg suppression appears to be due to the resistance of Teff in both T1D patients and NOD mice [32,35]. In the current study, we found both higher frequency of Treg and lower proliferation of Tcon in PLN from young recipients, suggesting that the increased Treg are functionally effective. We cannot rule out the possibility of hypo-function of Treg, for example in homing to lymph nodes, in older compared to young recipients in our experiments. However, D'Alise et. al. [35] demonstrated comparable suppressive function of Treg from NOD mice at 6w or 12-20w to Treg from B6g7 at same age. Similarly, Mellanby et. al. [33] reported that Treg from NOD mice at 7w and 16w comparably suppress the proliferation of NOD Teff. D'Alise et. al. also showed reduced NOD T effector susceptibility to suppression [35].Taken together, available data argue that PLN Treg abundance is key to the difference in post-transplantation survival between young and older recipients in our current study. In addition to prevention of diabetogenesis, the increased PLN Treg from young recipients may also promote beta cell regeneration [36].

Our results correlated with our previous finding showing that increased residual Tregs in recipients block BM-HSC-mediated transfer of T1D from BDC2.5NOD donors [37]. Similarly, evidence suggests that higher percentages of Treg cells in PLN, spleen and peripheral blood mediate the resistance of diabetes in aged NOD mice [38]. It is well established that diabetes incidence in NOD mice housed in specific pathogen-free facilities is higher than those in “dirty” facilities [39,40], and, diabetes in NOD mice is prevented by administration of complete Freund’s adjuvant, which is mainly composed of mycobacteria [41,42]. One mechanism proposed for these protective scenarios is the enhancement of pancreatic lymph node Treg [43]. Indeed, increases in PLN Treg by other infections/treatments, such as Coxsackie virus B3 or lymphocytic choriomeningitis virus [44], TLR2 agonist [45], and parasitic helminth [46] also are associated with protection of NOD mice from T1D. Thus, higher abundance of Treg may be a general mechanism underlying the resistance of diabetes in mulitiple circumstances. Treg in PLN from established T1D lose their immunosuppressive function [47]. A progressive decline of Treg in inflamed islets was observed in NOD mice [48]. In our setting, more advanced disease in older recipients may be linked to more inflammation in PLN than young recipients. Pro-inflammatory IL-12, IL-1β, IL-6 and IL-17 cytokines lead to the loss of Treg from T1D patients [49–52]. More pro-inflammatory cytokines from older vs. young recipients may similarly reduce PLN Treg.

Peripheral reduction of Treg in the local environment instead of centrally diminished Treg development in thymi of older recipients was confirmed by our finding of comparable thymic Treg distribution in the young and older groups (Fig 4). However, our studies did not precisely identify the site of origin of the increased Treg in PLN of young recipients. It would be of interest to use markers such as Helios to distinguish nTreg from induced aTreg. Notably, in MLN and blood, Tcon from young recipients also proliferated less than those from older recipients, although no difference of MLN or blood Treg distribution for those two groups was observed (Fig 3). Recent literature demonstrated MLN as a place for extrathymic development of murine T cells post BM transplantation [53], suggesting that MLN may also be a source of Tcon in PLN in our system, using blood as a bridge. Although Tcon proliferated less in PLN, MLN and blood from young recipients than from older recipients, the percentage of Tcon was similar for the two groups (Figs 2 and 3). A difference in Tcon apoptosis in young vs. older recipients may explain this discrepancy, which is an interesting direction for future studies.

In conclusion, we observed more rapid progression of T1D post-transplantation in older NOD mouse recipients with advanced disease than in young recipients at early stage of disease, although both groups received BM-HSC from the same syngeneic donors, and both groups had normal blood glucose at transplantation. Lower abundance of Treg and higher proliferation of Tcon found in PLN from the older recipients may drive the rapid progression of T1D in the older group post BM-HSC transplantation. The results provide new evidence supporting HSC transplantation at young age/earlier rather than at older age/later disease stage. Here, we used the classic myoablative (lethal irradiation) murine conditioning regimen pre-transplant. Clinically, although high-dose total body irradiation may improve efficiency of bone marrow transplantation therapy in treating type 1 diabetes [54] and is reported to be safe for children with acute myeloid leukemia in first remission [55] and other leukemia [56] even at age <2 years [57], side effects of this conditioning regimen, including increased tumor risk and infertility, need to be considered [58].

## Materials and Methods

### Ethics Statement

NOD mice were first purchased from the Jackson Laboratory (Bar Harbor, ME, USA) then bred and housed in the Stanford Veterinary Service Center. Animal research ethics committee of Stanford University-Administrative Panel for Laboratory Animal Care (APLAC) approved all the methods used in this study under the protocol numbered 15867. Mice with blood glucose higher than 450mg/dl were sacrificed immediately by CO2 exposure and cervical dislocation to avoid further end-stage diabetes suffering.

### BM-HSC preparation

BM cells were harvested from femurs and tibias of 3- to 5-month old female NOD mice (50% of which had high blood glucose (>250mg/dl)). The donor selection criterion is based on the phenomenon that about 50% mice at our facility get diabetes at 3–5m, thus, to mimic this situation, we included 50% of diabetic and 50% of non-diabetic mice as our donors. ckit^+^ BM cells were enriched using anti-CD117 microbeads then stained with monoclonal antibodies for linage (Lin) markers (CD3, CD4, CD8, B220, Gr1, Mac1, Ter119) and stem/progenitor cell markers (ckit and Sca-l). HSC (cKit^+^Sca-1^hi^Lin^-^) were obtained by sorting, using a FACS-Aria (BD Bioscience, San Jose, CA, USA) [37,59,60]. Antibodies were purchased from eBioscience (San Diego, CA, USA).

### BM-HSC transplantation

Female NOD mice at 4–6w or 3m with normal blood glucose were lethally irradiated at 980 cGy (in two doses with 4h intervals). The irradiated recipients were transplanted with ten thousand HSC per mouse by tail vein injection [37,59,60]. Donor and recipient derived cells were distinguished by different CD45 subtype expression, i.e. CD45.1 vs. CD45.2. Irradiation-only control mice without transplantation all died within 2w.

### Measurement of T1D progression

T1D development in syngeneic BM-HSC NOD recipients was monitored by blood glucose level. The glucose level in one drop of tail vein blood was checked with the One-Touch Ultra glucose meter (Life Scan Inc., Milpitas, CA, USA [37]). Recipient mice with blood glucose concentrations of <200mg/dl were considered eu-glycemic or non-diabetic, whereas those with blood glucose concentrations of >450mg/dl were defined as end-stage diabetic and were sacrificed immediately for FACS analysis.

### Flow cytometric (FACS) analysis

Spleen, blood, pancreatic lymph nodes (PLN), mesenteric lymph nodes (MLN) and thymus from each BM-HSC recipient were harvested and lyzed with red blood cell lysis buffer when necessary. To analyze the percentage and proliferation of CD4^+^ T cell subsets, 2×10^6^ cells were first stained with antibodies recognizing surface markers CD4 and CD25 then fixed, permeabilized, and stained with the antibodies against Foxp3 and Ki67 (eBioscience). T conventional cells (Tcon) and regulatory T cells (Treg) were defined as CD4^+^CD25^−^Foxp3^-^ and CD4^+^ Foxp3^+^ respectively. To measure the I-A^g7^ level in defined antigen presenting cells (APC) subsets, 1×10^6^ cells were stained with antibodies recognizing different cell lineage markers: B220^+^ for B cells; CD11b^+^F4/80^+^ for macrophages; CD11b^+^F4/80^−^ for monocytes; and CD11c^+^ for dendritic cells (DC) together with the antibody against I-A^g7^. For intracellular staining of cytokines, 1×10^6^ spleen cells were stimulated with Leukocyte Activation Cocktail (BD Biosciences) for 4h at 37°C followed by surface staining of CD4 and CD25 then intracellular staining with antibodies specific for Foxp3, IFNγ (eBioscience) and IL-17. Stained cells were analyzed on a LSR II flow cytometer (BD Bioscience), and data were analyzed using Flowjo software (Tree Star Inc., Ashland, OR, USA). Antibodies were purchased from Biolegend (San Diego, CA, USA), unless specified otherwise.

### Statistical analyses

The relationship between the age of NOD recipients at transplantation and the progression of T1D was analyzed with Chi-square test. The log-rank (Mantel-Cox) test was used to compare survival rates between young and older recipients. Mice with blood glucose >450mg/dl were counted as deceased. Other comparisons for T cell subset distribution and proliferation, cytokine production of Tcon and I-A^g7^ levels on APC between older and young recipients were analyzed with unpaired 2-tailed T test. All statistical analyses were done with Prism software (GraphPad Software, Inc., La Jolla, CA). P <0.05 was considered statistically significant.

## Supporting Information

S1 Fig The percentages and absolute numbers of Treg and Tcon in pancreatic lymph nodes (PLN) of non-transplanted (NT) control NOD mice at different ages. PLN from NT NOD mice at different ages, i.e. 4–6w and 3m with normal blood glucose (NG) as the controls for transplant recipients at transplant as well as 3–4m and 7–8m with normal glucose (NG) or high blood glucose (HG, glucose >450mg/dl) as controls for rapid progressors and resistants post-transplantation were harvested. Six mice were used for each (age + NG/HG) group. Same staining pattern and gating strategy were used as in Fig 2. The percentage and absolute numbers of Tcon (panels A and C) and those of Treg (panels B and D) were shown and analyzed using paired two-tailed T test. No statistical significance was found in either Treg or Tcon populations among different ages with same glucose level or between age-matched NG and HG groups.
(TIFF)

S2 Fig Proportions, proliferation and cytokine production in CD8^+^ T cells from central and peripheral immune organs of older rapid progressors and young resistants. The proportions and proliferation levels of CD8^+^ T cell data from thymus (Thy, panel E) and peripheral immune organs including pancreatic lymph nodes (PLN, panel A), spleen (Sp, panel B), mesenteric lymph nodes (MLN, panel C), and blood (BLD, panel D) of older rapid progressors (as older, n = 6) vs. young resistants (as young, n = 5) are shown. As in CD4^+^ T cells, CD8^+^ T cells were divided into CD25^-^Foxp3^-^ and Foxp3^+^ populations. Ki67 levels were used to detect the proliferation of those different populations. Cytokines (IFNγ and IL-17) production by splenic CD8^+^ T cells is shown in the panel F. No statistical significance was found in proportions and proliferation of central or peripheral CD8^+^ T cells or in their cytokine production, comparing young resistants and older progressors by unpaired 2-tailed T test.
(TIFF)
