# Supplementary figures and images for: Immunological Basis for Rapid Progression of Diabetes in Older NOD Mouse Recipients Post BM-HSC Transplantation
Source: PLoS One. 2015 May 28;10(5):e0128494. doi: 10.1371/journal.pone.0128494 (PMC4447290; doi:10.1371/journal.pone.0128494)

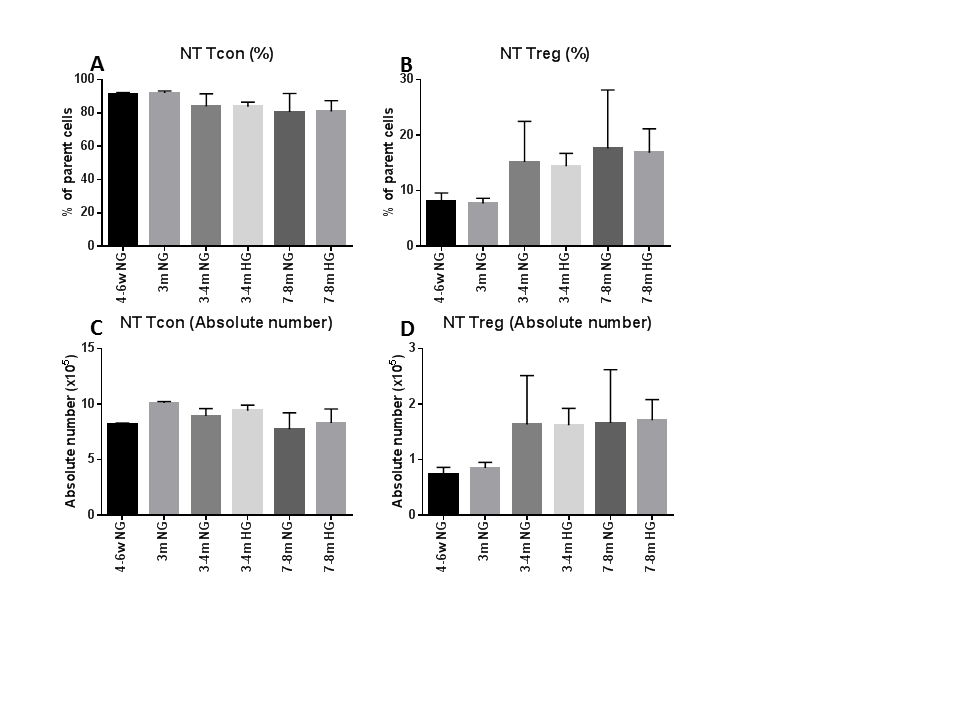

Supplement: S1 Fig — PLN from NT NOD mice at different ages, i.e. 4–6w and 3m with normal blood glucose (NG) as the controls for transplant recipients at transplant as well as 3–4m and 7–8m with normal glucose (NG) or high blood glucose (HG, glucose >450mg/dl) as controls for rapid progressors and resistants post-transplantation were harvested. Six mice were used for each (age + NG/HG) group. Same staining pattern and gating strategy were used as in Fig 2. The percentage and absolute numbers of Tcon (panels A and C) and those of Treg (panels B and D) were shown and analyzed using paired two-tailed T test. No statistical significance was found in either Treg or Tcon populations among different ages with same glucose level or between age-matched NG and HG groups. (TIFF) [file pone.0128494.s001.tiff]

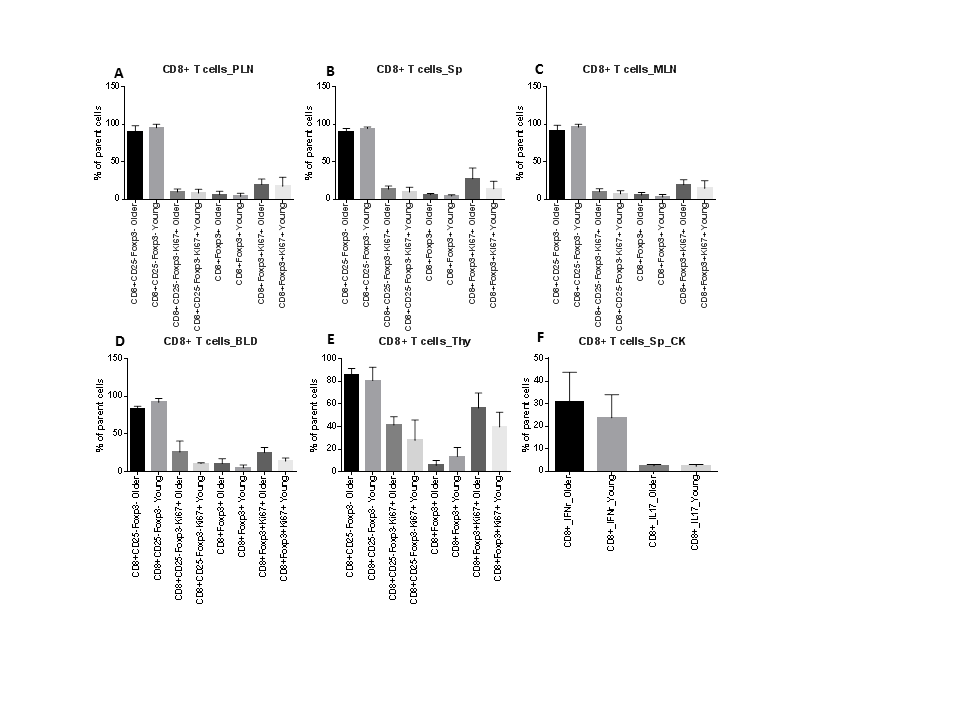

Supplement: S2 Fig — The proportions and proliferation levels of CD8+ T cell data from thymus (Thy, panel E) and peripheral immune organs including pancreatic lymph nodes (PLN, panel A), spleen (Sp, panel B), mesenteric lymph nodes (MLN, panel C), and blood (BLD, panel D) of older rapid progressors (as older, n = 6) vs. young resistants (as young, n = 5) are shown. As in CD4+ T cells, CD8+ T cells were divided into CD25-Foxp3- and Foxp3+ populations. Ki67 levels were used to detect the proliferation of those different populations. Cytokines (IFNγ and IL-17) production by splenic CD8+ T cells is shown in the panel F. No statistical significance was found in proportions and proliferation of central or peripheral CD8+ T cells or in their cytokine production, comparing young resistants and older progressors by unpaired 2-tailed T test. (TIFF) [file pone.0128494.s002.tiff]
